# Prevalence and predictors of taking tetanus toxoid vaccine in pregnancy: a cross-sectional study of 8,722 women in Sierra Leone

**DOI:** 10.1186/s12889-020-08985-y

**Published:** 2020-06-05

**Authors:** Sanni Yaya, Komlan Kota, Amos Buh, Ghose Bishwajit

**Affiliations:** 1 Faculty of Social Sciences, School of International Development and Global Studies, University of Ottawa, 120, University Private, Ottawa, ON K1N 6N5 Canada; 2 The George Institute for Global Health, The University of Oxford, Oxford, UK; 3 Faculty of Health Sciences, Interdisciplinary School of Health Sciences, University of Ottawa, Ottawa, Ontario Canada

**Keywords:** Tetanus toxoid, Immunization, Pregnant women, Global health, Prevalence, Predictors, Multiple Indicator cluster survey, Sierra-Leone

## Abstract

**Background:**

Immunization of women during pregnancy to protect them and their infants against tetanus, pertussis and influenza is recommended by the World health Organization (WHO). However, there is limited information about the coverage rate and associated factors in low-income countries. The aim of this study was to measure the prevalence and predictors of taking tetanus toxoid among pregnant women in Sierra Leone.

**Methods:**

This study was based on the fifth round of Multiple Indicator Cluster Survey (MICS 5) conducted in Sierra Leone in 2017. In total 8722 women aged between 15 and 49 years were included in this study. Outcome variable was taking of Tetanus Toxoid vaccination during the last pregnancy. Data were analyzed using cross-tabulation and logistic regression methods.

**Results:**

The overall prevalence of receiving TT immunization during women’s last pregnancy was 96.3% and that of taking at least two doses was 82.12%. In the regression analysis, women from Mende ethnicity had a 0.48 fold lower chance of being immunized (OR = 0.480, 95% CI = 0.385,0.59768) than those from the other ethnicity. In addition, women who attended at least four ANC visits had higher odds of receiving TT vaccine (OR = 1.919, 95% CI = 1.639,2.245) compared to those who attended less ANC visits. Stratified by areas, this association was observed in both urban (OR = 2.661, 95% CI = 1.924,3.679) and rural areas (OR = 1.716, 95% CI = 1.430,2.059). Attending at least four ANC visits showed a positive association with receiving at least two doses TT (OR = 2.434, 95% CI = 1.711,3.464) in both urban (OR = 2.815, 95% CI = 1.413,5.610) and rural areas (OR = 2.216, 95% CI = 1.463,3.356) as well.

**Conclusion:**

Higher number of ANC visits, mass media exposure and higher wealth quintile increased the odds of receiving TT immunization. In addition, minimum two doses which were identified to reduce neonatal mortality. Therefore, immunization campaigns targeting improved utilization of healthcare and immunization services by women of childbearing age in Sierra Leone are strongly recommended.

## Background

Tetanus is a life-threatening bacterial infection caused by the contamination of wounds with *Clostridium tetani* and is characterized by muscle spasms and autonomic nervous system dysfunction [1, 2]. While the disease affects people of all ages, it is particularly common and deadly in neonates [3, 4]. In 2017 for example, WHO estimated 30,848 newborns died from neonatal tetanus (NT), which represented 85% reduction form the situation in 2000 [5]. Most of these deaths take place in developing countries, especially in rural communities where childbirth and abortion were practiced with poor hygienic conditions [6–8].

Preventative measures for this disease have long been established with the use of the Tetanus toxoid (TT) vaccine which is one of the most effective and protective against maternal tetanus deaths and has been recommended by WHO in many countries [9]. For this reason, WHO, since 2006, has provided guidelines for the immunization of pregnant women against tetanus [9–12]. The guidelines require that a pregnant woman receives three doses of tetanus vaccine to protect her and her newborn(s) from tetanus, compared to those whose woman do not receive the vaccine [13]. Two or more doses of tetanus toxoid vaccine in pregnancy affords the fetus passive immunity and have been shown to reduce NT mortality by 96% [14, 15].

Although much progress has been made in reducing deaths from maternal and neonatal tetanus during the past two decades [16], it remains a public health problems and poses a viable threat in many developing countries, mostly in Asia and Africa [1, 17, 18]. Some of the reasons for the persistence of TT include: low income, poor access to antenatal care, lack of education, lack of information, unskilled birth attendants, and unhygienic and traditional abortion practices [16, 19, 20].

In 2011, Sierra Leone was listed as one of 38 countries where maternal and neonatal mortality is still a public health problem [21]. The country topped 4 years after the list of countries with high maternal mortality ratio with an estimate of 1360 maternal deaths per 100,000 live births [22, 23]. Despite this, most deliveries in Sierra Leone are still conducted at home by unskilled birth attendants, making the country one of the most dangerous places for a woman to be pregnant and a child to be born [21]. Therefore, over the years, WHO, UNICEF, UNFPA and its partners strongly urge women throughout the country to take TT vaccine to eliminate maternal and neonatal tetanus [20, 24]. In this study, we assessed the prevalence and predictors of taking the tetanus toxoid vaccine among pregnant women in Sierra Leone using data from the country’s Multiple Indicator Cluster Survey (MICS) for 2017.

## Methods

### Data source

The present study used data from the Sixth round of the Multiple Indicator Cluster Survey (MICS), done in Sierra Leone in 2017. Statistics Sierra Leone conducted this survey with technical support from the United Nations Children’s Fund (UNICEF). The purpose of the survey was to generate quality data on maternal and child health that will enhance evidence-based policy making and program monitoring towards the Sustainable Development Goals. Data for the survey was collected between May and August 2017 and the sample frame of the 2015 Sierra Leone Population and Housing Census was used. The primary sampling units (PSUs) selected at the first stage were census enumeration areas (EAs). A new listing of households was conducted in each sample EA, and the sample households were selected at the second stage [25].

The 2017 Sierra Leone MICS survey is a representative household surveys and the selected samples reflect the populations from which they are drawn. The survey was designed to have sufficient sample to be representative at the national level and first subnational level (e.g., region). A multi-stage, stratified cluster sampling approach was used for the selection of the survey sample.

The survey included 18,006 women aged 15–49 years old, 17,873 of whom were interviewed (giving a response rate of 99.3%). More information about the survey have been published elsewhere [25, 26].

### Variables

The outcome variable of interest for the present study was adequate use of Tetanus toxoid (TT) which was assessed by asking the women whether or not they received TT vaccination during their last pregnancy. Following WHO recommendations, receiving at least two doses of TT is considered as adequate dose of TT while less than two (< 2) doses are regarded as inadequate.

Explanatory variables were selected based on their demonstrated association in previous studies such as systematic reviews on determinants of effective vaccine coverage in low and middle-income countries [26].

The following independent variables were used in our analysis due to their known /theoretical association with utilization of TT immunization in the general population:

Age groups, residency, region, education, ethnicity, wealth status, parity, radio use, TV use, Internet use, Has mobile and ANC visit [20, 27–29].

### Statistical analyses

We analyzed our data using Stata version 14 software. We used the survey command (svy) to account for survey weights and computed prevalence rates of receiving TT immunization for each explanatory variable as percentages. To assess the correlates of TT immunization, logistic regression models were used to compute the odds ratios of the associations between taking TT immunization and the covariates. The analyses were carried out to examine the crude and adjusted association respectively. All variables found significant in the bivariate analysis were included in the model. We also performed variance inflation factor (VIF) after the regression models to check for multicollinearity. This is useful as it helps estimate the extent to which the variance of a regression coefficient is inflated due multicollinearity among our predictors. In our case, we found no indication of multicollinearity as the VIF mean value for all covariates in our study is 1.17. For all analysis, statistical significance was set at a *p*-value of < 0.05.

## Results

### Descriptive analysis

The Table 1 shows the sample characteristics and the prevalence of taking tetanus toxoid. Of 8722 potentially eligible women aged between 15 and 49 years, 96.32% reported receiving TT immunization and 65.16% reported receiving at least two doses of TT immunization during their most recent pregnancy. Among women reported receiving TT immunization, one in four (24.7%) were 25–29 years of age, two-third lived in rural areas (68.7%), about two-fifth were from north region (38.8%), three-fifth had no formal education (59.7%), 35.5% were from Mende ethnicity, 27.1% were from households with the poorest wealth quintile, and 46.2% had delivered at least once. It also shows that the proportion of taking TT immunization as higher among pregnant women who did not use radio (61.9%), not watched TV (83.2%), not have access of internet (96.8%), not use a mobile phone (64.2%), as well as women who attended at least three ANC visits during their last pregnancy (85.5%). Also, high proportion of women who received at least two doses of TT immunization were noted among women aged 25–29 years (24.8%, those who lived in rural areas (59.4%), came from north region (35.3%), had no formal education (54.8%), participants who from the mende ethnicity (33.4%), those from households with the poorest quintile (22.2%),and women who had delivered at least once (47.7%). Concerning mass media, a large majority of women who reported receiving two doses of TT immunization did not use of radio, TV, internet and mobile respectively (58.1, 75.7, 95.7 and 59.8%). Interestingly, 88.5% of pregnancy women who reported receiving two doses of TT immunization had made at least three ANC visits.

**Table 1:** Prevalence of taking tetanus toxoid in Sierra Leone (*n* = 8722)

		Took tetanus toxoid 96.32%			At least 2 doses of tetanus toxoid 82.12%		
	N; %	No	Yes	*p*-value	No	Yes	*p*-value
**Age groups**							
15–19	809; 9.3	10.5 (6.9,15.6)	8.8 (8.1,9.5)	0.015	8.4 (7.0,10.1)	8.9 (8.1,9.7)	.009
20–24	2055; 23.6	24.5 (19.1,31.0)	24.1 (23.0,25.2)		25.3 (22.7,28.2)	23.8 (22.6,25.0)	
25–29	2151; 24.7	19.9 (15.4,25.2)	24.8 (23.8,25.9)		24.4 (21.9,27.2)	24.9 (23.7,26.1)	
30–34	1664; 19.1	18.1 (14.0,23.0)	19.4 (18.5,20.5)		19.9 (17.6,22.3)	19.4 (18.3,20.5)	
35–39	1300; 14.9	18.6 (13.3,25.3)	14.7 (13.9,15.6)		14.4 (12.3,16.8)	14.8 (13.9,15.8)	
40–44	536; 6.1	5.7 (3.6,9.1)	5.8 (5.3,6.4)		5.7 (4.5,7.1)	5.9 (5.3,6.5)	
45–49	207; 2.4	2.7 (1.2,5.9)	2.3 (2.0,2.7)		1.9 (1.2,2.9)	2.4 (2.1,2.9)	
**Residency**							
Urban	2727; 31.3	34.9 (28.0,42.4)	40.6 (39.2,42.1)	.003	48.8 (45.6,52.0)	38.8 (37.3,40.4)	.061
Rural	5995; 68.7	65.1 (57.6,72.0)	59.4 (57.9,60.8)		51.2 (48.0,54.4)	61.2 (59.6,62.7)	
**Region**							
East	1931; 22.1	9.8 (6.8,14.1)	23.5 (22.4,24.7)	<.001	10.5 (8.8,12.4)	26.4 (25.2,27.7)	<.001
North	3384; 38.8	50.7 (44.0,57.4)	35.3 (34.1,36.6)		45.5 (42.4,48.7)	33.1 (31.7,34.5)	
South	2131; 24.4	17.9 (13.8,22.9)	19.3 (18.4,20.3)		10.2 (8.8,11.7)	21.3 (20.2,22.5)	
West	1276; 14.6	21.6 (15.2,29.7)	21.8 (20.5,23.2)		33.8 (30.6,37.2)	19.2 (17.8,20.6)	
**Education**							
Pre-Primary Or None	5210; 59.7	63.2 (56.0,69.8)	54.8 (53.5,56.1)	<.001	53.5 (50.4,56.6)	55.1 (53.7,56.5)	<.001
Primary	1180; 13.5	16.1 (11.3,22.6)	13.6 (12.8,14.5)		12.6 (10.8,14.7)	13.9 (12.9,14.8)	
Lower Secondary	1326; 15.2	10.5 (7.1,15.1)	16.4 (15.5,17.4)		16.9 (14.6,19.5)	16.3 (15.3,17.4)	
Upper Secondary/higher	1006; 11.5	10.3 (6.1,16.7)	15.1 (14.1,16.2)		16.9 (14.5,19.8)	14.7 (13.6,15.9)	
**Ethnicity**							
Mende	3100; 35.5	19.1 (14.0,25.5)	33.4 (32.2,34.7)	<.001	16.0 (14.0,18.3)	37.2 (35.8,38.6)	<.001
Temne	2628; 30.1	44.7 (38.2,51.4)	32.3 (31.0,33.6)		47.4 (44.2,50.7)	29.0 (27.6,30.4)	
Limba	639; 7.3	5.1 (2.9,8.9)	8.0 (7.2,8.7)		12.2 (10.4,14.4)	7.0 (6.3,7.8)	
Other	2355; 27.0	31.1 (25.3,37.5)	26.3 (25.1,27.6)		24.3 (21.6,27.2)	26.8 (25.5,28.1)	
**Wealth status**							
Poorest	2361; 27.1	24.5 (19.7,29.9)	22.2 (21.2,23.2)	<.001	19.3 (17.3,21.5)	22.8 (21.7,23.9)	<.001
Second	2110; 24.2	25.6 (20.7,31.2)	21.1 (20.1,22.1)		17.9 (15.9,20.1)	21.8 (20.7,23.0)	
Middle	1922; 22.0	20.6 (15.6,26.6)	20.4 (19.3,21.5)		18.7 (16.5,21.1)	20.7 (19.6,22.0)	
Fourth	1317; 15.1	16.8 (11.7,23.6)	19.0 (17.8,20.2)		22.7 (19.9,25.9)	18.2 (16.9,19.5)	
Richest	1012; 11.6	12.6 (8.2,18.9)	17.3 (16.2,18.6)		21.4 (18.5,24.5)	16.4 (15.2,17.7)	
**Parity**							
1/2	4028; 46.2	47.5 (40.9,54.3)	47.7 (46.4,48.9)	<.001	47.5 (44.4,50.6)	47.7 (46.4,49.1)	<.001
3/4	2744; 31.5	28.2 (22.7,34.4)	30.8 (29.7,32.0)		32.7 (29.9,35.6)	30.4 (29.2,31.7)	
5 > 4	1950; 22.4	24.3 (19.1,30.4)	21.5 (20.5,22.5)		19.8 (17.6,22.3)	21.8 (20.8,22.9)	
**Radio use**							
Not At All	5398; 61.9	70.4 (63.9,76.1)	58.1 (56.8,59.4)	.023	58.1 (55.0,61.2)	58.1 (56.7,59.5)	.035
Less Than Once A Week	2354; 27.0	21.0 (16.0,27.1)	29.3 (28.1,30.5)		29.5 (26.6,32.5)	29.2 (27.9,30.6)	
Almost Every Day	970; 11.1	8.6 (5.7,12.8)	12.6 (11.8,13.6)		12.4 (10.4,14.8)	12.7 (11.7,13.7)	
**TV use**							
Not At All	7183; 82.4	83.2 (76.8,88.1)	75.7 (74.4,76.9)	<.001	72.2 (68.9,75.3)	76.5 (75.1,77.8)	<.001
Less Than Once A Week	1005; 11.5	10.2 (6.5,15.8)	14.9 (13.9,16.0)		16.5 (14.0,19.2)	14.6 (13.5,15.7)	
Almost Every Day	534; 6.1	6.5 (3.6,11.5)	9.4 (8.5,10.3)		11.3 (9.1,14.0)	8.9 (8.0,9.9)	
**Internet use**							
Yes	280; 3.2	2.5 (1.0,5.7)	4.3 (3.7,4.9)	<.001	5.5 (4.1,7.4)	4.0 (3.4,4.7)	<.001
No	8442; 96.8	97.5 (94.3,99.0)	95.7 (95.1,96.3)		94.5 (92.6,95.9)	96.0 (95.3,96.6)	
**Has mobile**							
Yes	3121; 35.8	20.7 (15.5,27.0)	40.2 (38.9,41.5)		43.2 (40.1,46.3)	39.6 (38.2,41.0)	<.001
No	5601; 64.2	79.3 (73.0,84.5)	59.8 (58.5,61.1)		56.8 (53.7,59.9)	60.4 (59.0,61.8)	
**ANC visit**							
Inadequate	1070; 12.3	27.8 (20.4,36.6)	11.5 (10.7,12.3)	<.001	17.9 (15.5,20.4)	10.1 (9.3,10.9)	<.001
Adequate	7456; 85.5	72.2 (63.4,79.6)	88.5 (87.7,89.3)		82.1 (79.6,84.5)	89.9 (89.1,90.7)	

## Regression analysis

Table 2 shows the results of regression analysis on the predictors of tetanus toxoid use among urban and rural pregnant women. In the logistic regression model including the variables with a *p* < 0.05, *p* < 0.01, *p* < 0.001 in the univariate analysis, women’s age, region, educational level, ethnicity, wealthy, parity, mass media, and number of ANC visits were found to be associated with TT immunization (Table 2). Our analysis revealed that women from Mende ethnicity had a 0.48 fold lower chance of being immunized (OR = 0.480, 95% CI = 0.385,0.59768) than those from the other ethnicity. We found also having higher wealth quintile was significantly associated with higher odds of receiving TT immunization (OR = 1.509, 95% CI = 1.071,2.126) compared to those with the poorest wealth quintile. In addition, women who attended at least four ANC visits had higher odds of receiving TT vaccine (OR = 1.919, 95% CI = 1.639,2.245) compared to those who attended less ANC visits. Stratified by areas, this association was observed in both urban (OR = 2.661, 95% CI = 1.924,3.679) and rural areas (OR = 1.716, 95% CI = 1.430,2.059).

**Table 2:** Predictors of tetanus toxoid use among urban and rural women in Sierra Leone, 2017

	Total	Urban	Rural
**Age (15–19)**			
20–24	1.020	0.882	1.115
	[0.811,1.283]	[0.585,1.330]	[0.841,1.478]
25–29	1.104	1.079	1.124
	[0.867,1.406]	[0.696,1.673]	[0.837,1.508]
30–34	0.951	1.072	0.912
	[0.729,1.240]	[0.661,1.739]	[0.661,1.258]
35–39	1.123	1.117	1.141
	[0.842,1.498]	[0.659,1.892]	[0.806,1.614]
40–44	1.133	1.501	1.050
	[0.800,1.604]	[0.751,3.000]	[0.698,1.579]
45–49	1.412	1.885	1.273
	[0.879,2.268]	[0.674,5.274]	[0.742,2.183]
**Residence (Urban)**			
Rural	1.045		
	[0.842,1.295]		
**Region (East)**			
North	0.438^***^	0.443^***^	0.433^***^
	[0.349,0.550]	[0.289,0.679]	[0.329,0.569]
South	0.463^***^	0.953	0.396^***^
	[0.371,0.579]	[0.556,1.634]	[0.305,0.513]
West	0.255^***^	0.327^***^	0.137^***^
	[0.195,0.335]	[0.220,0.485]	[0.078,0.243]
**Education (Primary)**			
Secondary	1.043	1.020	1.014
	[0.864,1.260]	[0.750,1.387]	[0.794,1.294]
Higher	0.885	0.789	1.137
	[0.698,1.122]	[0.572,1.087]	[0.735,1.757]
**Ethnicity (Other)**			
Mende	0.480^***^	0.431^***^	0.483^***^
	[0.385,0.597]	[0.299,0.620]	[0.364,0.641]
Temne	0.452^***^	0.536^**^	0.378^***^
	[0.344,0.594]	[0.339,0.845]	[0.266,0.536]
Limba	0.747^**^	0.709	0.721^*^
	[0.605,0.923]	[0.494,1.019]	[0.552,0.943]
**Wealth (Poorest)**			
Second	1.106	2.291	1.085
	[0.937,1.306]	[0.863,6.079]	[0.916,1.286]
Middle	1.065	1.873	1.020
	[0.890,1.275]	[0.868,4.044]	[0.842,1.235]
Fourth	1.387^*^	2.090	1.557
	[1.055,1.824]	[0.980,4.457]	[0.949,2.552]
Richest	1.509^*^	2.055	3.462^*^
	[1.071,2.126]	[0.933,4.530]	[1.311,9.145]
**Parity (1/2)**			
3/4	0.875	0.844	0.860
	[0.748,1.022]	[0.637,1.117]	[0.711,1.039]
> 4	0.921	0.732	0.972
	[0.754,1.125]	[0.495,1.084]	[0.768,1.231]
**Radio (Never)**			
Few days a week	1.206	1.292	1.210
	[0.992,1.466]	[0.954,1.751]	[0.930,1.574]
Almost everyday	1.029	1.270	0.844
	[0.834,1.268]	[0.911,1.771]	[0.641,1.112]
**TV (Never)**			
Few days a week	0.878	0.882	0.722
	[0.665,1.160]	[0.632,1.230]	[0.411,1.269]
Almost everyday	0.922	0.950	0.479
	[0.669,1.270]	[0.664,1.359]	[0.190,1.208]
**Internet (Yes)**			
No	1.167	1.382	0.688
	[0.839,1.622]	[0.940,2.032]	[0.321,1.473]
**Has mobile (No)**			
Yes	1.348	1.486	0.890
	[0.957,1.900]	[0.986,2.240]	[0.466,1.700]
**ANC visit (Inadequate)**			
Adequate	1.919^***^	2.661^***^	1.716^***^
	[1.639,2.245]	[1.924,3.679]	[1.430,2.059]

N.B. Odds ratios; 95% confidence intervals in brackets

^*^p < 0.05, ^**^p < 0.01, ^***^p < 0.001

Table 3 shows the predictors of receiving at least two doses of TT among urban and rural women by using the regression analysis. The odds of receiving TT immunization didn’t vary educational level. Women from Mende ethnicity were 0.351 times (95% CI = 0.205,0.603) as likely as others to receive two doses of TT immunization. Lastly, those who attended at least four ANC visits had higher odds of receiving at least two doses TT (OR = 2.434, 95% CI = 1.711,3.464) in both urban (OR = 2.815, 95% CI = 1.413,5.610) and rural areas (OR = 2.216, 95% CI = 1.463,3.356).

**Table 3:** Predictors of taking adequate dose of tetanus toxoid among urban and rural women in Sierra Leone, 2017

	Total	Urban	Rural
**Age (15–19)**			
20–24	1.139 [0.669,1.940]	1.680 [0.664,4.250]	1.009 [0.521,1.955]
25–29	1.379 [0.772,2.466]	2.697 [0.914,7.956]	1.116 [0.551,2.259]
30–34	1.516 [0.774,2.971]	2.212 [0.656,7.466]	1.302 [0.571,2.972]
35–39	1.214 [0.601,2.452]	1.659 [0.466,5.912]	1.076 [0.456,2.540]
40–44	2.003 [0.757,5.299]	1.104 [0.241,5.067]	3.145 [0.801,12.346]
45–49	1.949 [0.529,7.178]	1.745 [0.162,18.766]	1.927 [0.395,9.408]
**Residence (urban)**			
Rural	1.053 [0.609,1.822]		
**Region (East)**			
North	1.108 [0.652,1.883]	0.312^*^ [0.103,0.939]	1.496 [0.829,2.699]
South	0.747 [0.442,1.261]	2.157 [0.437,10.641]	0.548^*^ [0.302,0.996]
West	0.879 [0.446,1.733]	0.392 [0.140,1.091]	1.025 [0.207,5.067]
**Education (Primary)**			
Primary	0.969 [0.596,1.574]	2.011 [0.784,5.157]	0.692 [0.393,1.217]
Secondary	1.443 [0.699,2.980]	1.245 [0.507,3.060]	5.054 [0.639,39.950]
**Ethnicity (Other)**			
Mende	0.351^***^ [0.205,0.603]	1.543 [0.665,3.582]	0.178^***^ [0.095,0.332]
Temne	0.910 [0.382,2.172]	2.737 [0.788,9.508]	0.567 [0.178,1.811]
Limba	0.668 [0.395,1.129]	3.636^**^ [1.376,9.610]	0.324^***^ [0.177,0.593]
**Wealth (Poorest)**			
Second	0.934 [0.609,1.431]	0.935 [0.176,4.959]	0.949 [0.613,1.468]
Middle	0.997 [0.622,1.597]	0.780 [0.291,2.093]	1.180 [0.698,1.994]
Fourth	0.925 [0.463,1.847]	0.971 [0.442,2.134]	0.553 [0.213,1.436]
Richest	0.978 [0.402,2.382]	1.151 [0.794,3.079]	1.027 [0.122,8.667]
**Parity (1/2)**			
3/4	1.175 [0.768,1.796]	1.117 [0.492,2.535]	1.216 [0.736,2.007]
> 4	0.938 [0.553,1.589]	1.117 [0.385,3.241]	0.926 [0.503,1.705]
**Radio (Never)**			
Few days a week	1.767^*^ [1.004,3.113]	2.389 [0.922,6.194]	1.641 [0.800,3.368]
Almost everyday	1.422 [0.851,2.377]	1.314 [0.600,2.877]	1.638 [0.797,3.365]
**TV (Never)**			
Few days a week	1.229 [0.512,2.950]	0.726 [0.254,2.075]	2.524 [0.341,18.681]
Almost everyday	0.616 [0.319,1.191]	0.570 [0.242,1.342]	0.550 [0.178,1.696]
**Internet (Yes)**			
No	0.646 [0.194,2.150]	0.736 [0.160,3.374]	0.867 [0.116,6.464]
**Has mobile (No)**			
Yes	0.644 [0.260,1.598]	1.071 [0.295,3.879]	0.281^*^ [0.080,0.990]
**ANC visit (Inadequate)**			
Adequate	2.434^***^ [1.711,3.464]	2.815^**^ [1.413,5.610]	2.216^***^ [1.463,3.356]

^*^p < 0.05, ^**^p < 0.01, ^***^p < 0.001

## Discussion

Literature reveals that immunization coverage among women of childbearing age is still a major public health problem especially developing countries. This study sought to assess the prevalence of both TT and adequate TT immunization, and their associated factors. Findings reveal that the proportion of pregnant women who received TT immunization was 97.8% and that of adequate doses was 82%. These findings concur with results from previous studies that reported higher prevalence of receiving TT immunization [30] but are inconsistent with studies that reported lower rates of receiving adequate doses of TT immunization [14, 19, 28, 29]. This difference may be due to cultural practices, and financial capabilities of women needed to seek immunization services.

We found in our regression model that maternal age, region of residence, wealth quintile, mass media, parity as well as ANC visits were significantly associated with higher odds of receiving TT immunization and that of adequate doses. For instance, we found that women aged 29–35 years old were associated with increased chances of receiving adequate doses of TT immunization compared to those aged 15–19 years. Similarly, evidence from Ethiopia, Ivory Coast and Turkey support our findings and have reported that women with advancing age were more likely to receive TT protective dose immunization than their counterpart [20, 30–32]. A possible reason for this could be that women with advanced age are more matured and might have had past exposure to knowledge about immunization and its benefits for themselves and their children.

This study also found that women from Mende ethnicity, compared to those form other ethnicity had higher odds of receiving TT immunization. This positive effect was only for rural women. Certain authors suggest that the ethnic difference could be due to lack of confidence, understanding and broader cultural factors about immunization among ethnic groups [32, 33].

To the best of our knowledge, our study is one of the first to consider the association between women’s reception of both TT immunization and taking adequate doses of TT and the women’s region of residence. We found that maternal TT immunization was significantly predicted by region where women lived. This positive association was true only for rural women living in the north region when compared to those from East region. Concerning the adequate doses of TT immunization, our study revealed that women living in the North and South region of Sierra Leonne had higher odds of having adequate doses of TT immunization than those who lived in the East region. We perceive that these regional differences could be due to regional imbalances in infrastructural development, education, healthcare service availability and access to information about vaccination.

In the current study, we also provided evidence household wealth quintile had no effect on receiving adequate doses of TT immunization when compared to those with the poorest wealth quintile. This finding is contrary to previous studies done in Ivory Coast and in Pakistan [26, 32, 34, 35]. We perceive that women from wealthy households have the financial means to seek healthcare services than those from poor households.

Also, this study found that being exposed to the mass media increased women’s demand for adequate doses of TT immunization. Hence, women who always listen to the radio, watch tv and who were in position of a mobile phone, had higher odds of receiving the adequate doses of TT immunization compared to those with no access to the mass media. This is similar to previous studies in which mass media played an important role in increasing the odds of receiving adequate doses of TT immunization [34, 36, 37]. Recently, studies conducted in Vietnam and in sub-Saharan Africa also supported our findings and reported that women having access to media were more likely to receive adequate dose of TT immunization than those with no access [38, 39]. We perceive that mass media devices such as the TV, radio and mobile phone assist in increasing health information to the population about immunization which influences immunization rate among women positively.

Concerning parity, it should be noted that women with higher parity had higher odds of receiving TT and also the adequate dose of TT immunization compared to those of lower parity. This findings agrees with results of previous studies conducted in Nigeria, Turkey, and Pakistan where TT immunization was found to be associated with higher parity [40–42]. We perceive that women with more children are used to receiving TT immunization as a routine during their antenatal care.

Interestingly, our study also showed that a higher number of ANC visits were strongly associated with higher odds of receiving TT immunization. Women who attended at least four ANC visits had higher odds of being immunized against tetanus than those who attended less. Regarding adequate doses of TT immunization, women who made at least four ANC visits during the last pregnancy had higher odds of receiving two doses of TT immunization compared to those who attended fewer visits. This finding is consistent with those of studies conducted in Ethiopia, Bangladesh, India and Laos [29, 37, 43, 44]. The plausible reason for this finding may be that women who have attended more ANC visits are more likely to be informed about the importance of TT immunization and would therefore receive it than those who attended less ANC visits.

However, the chances of receiving TT immunization and that of adequate doses were significantly lower in women with education, ethnicity, mass media used and parity. For instance, women with primary and higher educational level had lower odds of receiving TT immunization when compared to those with no formal education. This finding is in contrast with previous studies that reported TT immunization was positively associated with women’s level of education [14, 19, 29, 45–47].

Additionally, significantly lower TT immunization was found among women from the Temme ethnicity compared to those from another ethnicity. This observation was true only for urban women. Besides this, lower adequate doses of TT immunization were also found among women from the Limba ethnicity compared to those from another ethnicity. This negative association was true only for rural women but not for urban women. Possible explanation could be the lack of knowledge on the positive effects of TT immunization on maternal or fetal health [48].

Furthermore, women who were exposed to the mass media such as TV had lower chances of receiving TT immunization and that of adequate doses. This is consistent with cross-sectional studies conducted in Vietnam [39], China [49], Taiwan [50] and Canada [51], and inconsistent with studies done in Nepal [36] and Uganda [52]. Hearing negative information about immunizations in the mass media could be a possible barrier to be immunized.

### Strengths and limitations

This study is the first assess the prevalence and predictor of receiving TT immunization among women in Sierra Leone and therefore remains subject to reporting bias. One of the strengths of the study was the sample size which was relatively large and nationally representative**.** The data were collected from the most recent survey. However**,** this was a cross-sectional study which cannot be used to indicate causal inference of the association [53, 54] between pregnant women’s health and TT immunization.

## Conclusion

In this study, the prevalence rate of receiving TT immunization and that of adequate doses were higher among pregnant women in Sierra Leone. The findings from this study indicate that pregnant women with advancing age, who lived in the north and south region of Sierra Leonne, women from the Temba ethnicity, women with the highest household wealth quintile, women with higher parity, those who exposed to mass media such as radio, tv and mobile phone, as well as those who attended at least four ANC visits had higher odds of receiving TT immunization and that of adequate doses. However, women with primary and higher educational level had lower chances of receiving TT immunization when compared to those with no formal education. We recommend that immunization campaigns need to be conducted to improve healthcare and immunization service utilization by women of childbearing age.

## Data Availability

Data for this study were sourced from the UNICEF website: http://mics.unicef.org/

## Abbreviations

ANC: Antenatal care
CI: Confidence Interval
TT: Tetanus Toxoid
WHO: Word Health Organization
